# Casboundary: automated definition of integral Cas cassettes

**DOI:** 10.1093/bioinformatics/btaa984

**Published:** 2020-12-06

**Authors:** Victor A Padilha, Omer S Alkhnbashi, Van Dinh Tran, Shiraz A Shah, André C P L F Carvalho, Rolf Backofen

**Affiliations:** Institute of Mathematics and Computer Sciences, University of São Paulo, São Carlos, SP 13566-590, Brazil; Bioinformatics Group, Department of Computer Science, University of Freiburg, 79110 Freiburg, Germany; Bioinformatics Group, Department of Computer Science, University of Freiburg, 79110 Freiburg, Germany; COPSAC, Copenhagen University Hospitals Herlev and Gentofte, DK-2820 Gentofte, Denmark; Institute of Mathematics and Computer Sciences, University of São Paulo, São Carlos, SP 13566-590, Brazil; Bioinformatics Group, Department of Computer Science, University of Freiburg, 79110 Freiburg, Germany; Signalling Research Centres BIOSS and CIBSS, University of Freiburg, 79104 Freiburg, Germany

## Abstract

**Motivation:**

CRISPR-Cas are important systems found in most archaeal and many bacterial genomes, providing adaptive immunity against mobile genetic elements in prokaryotes. The CRISPR-Cas systems are encoded by a set of consecutive *cas* genes, here termed cassette. The identification of cassette boundaries is key for finding cassettes in CRISPR research field. This is often carried out by using Hidden Markov Models and manual annotation. In this article, we propose the first method able to automatically define the cassette boundaries. In addition, we present a Cas-type predictive model used by the method to assign each gene located in the region defined by a cassette’s boundaries a Cas label from a set of pre-defined Cas types. Furthermore, the proposed method can detect potentially new *cas* genes and decompose a cassette into its modules.

**Results:**

We evaluate the predictive performance of our proposed method on data collected from the two most recent CRISPR classification studies. In our experiments, we obtain an average similarity of 0.86 between the predicted and expected cassettes. Besides, we achieve *F*-scores above 0.9 for the classification of *cas* genes of known types and 0.73 for the unknown ones. Finally, we conduct two additional study cases, where we investigate the occurrence of potentially new *cas* genes and the occurrence of module exchange between different genomes.

**Availability and implementation:**

https://github.com/BackofenLab/Casboundary.

**Supplementary information:**

Supplementary data are available at *Bioinformatics* online.

## 1 Introduction

Prokaryotes face tremendous evolutionary pressures from viral predators, such as bacteriophages, which are responsible for eradicating almost half of the earth’s bacterial population each day (Suttle, 2016). This constant threat has been hypothesized to comprise the single most important driver of the planet life evolution (Koonin et al., 2020). Bacteria and archaea face an enormous incentive to defend themselves against viral invaders by evolving defense systems, some of which are innate and others adaptive. Clustered Regularly Interspaced Short Palindromic Repeats (CRISPRs) constitute one such nucleic acid based adaptive immune system, which functions through three distinct stages: acquisition, processing and interference. Upon a naive infection, a piece of viral nucleic acid is incorporated as a spacer between the repeats of the CRISPR locus on the host chromosome during its acquisition. The whole CRISPR locus, which includes memories from dozens of past viral infections, is transcribed into a long piece of RNA that is processed into small mature CRISPR RNAs (crRNAs), each corresponding to a different acquired viral epitope. crRNAs are loaded onto the Cas (Crispr ASsociated) interference complex, which then scans all intracellular nucleic acid for a matching nucleotide sequence, in which case the target nucleic acid is cleaved, effectively protecting the cell from reinfection by any virus for which a matching spacer exists.

Bacteriophages and archaeal viruses evade CRISPR immunity by several mechanisms. Known mechanisms include direct mutations of the nucleic acid such that it is no longer targeted by the host (Horvath et al., 2008), or the evolution of small anti-CRISPR proteins. These proteins interfere with the proper function of the Cas proteins that mediate CRISPR immunity by either clogging catalytic sites or preventing complex assembly. Hosts evade such anti-CRISPR immunity by carrying several distantly related CRISPR-Cas systems at once, and by frequently exchanging their CRISPR-Cas systems for different ones through horizontal gene transfer. This dynamic has driven the diversification of CRISPR-Cas systems into six types that are further subdivided into 33 subtypes (Makarova et al., 2020), each with its own evolutionary trajectory. Corresponding Cas protein subunits from two different hosts, even when belonging to the same subtype, can have sequences so distant that they are unalignable despite sharing the same underlying protein structure. Such extreme diversification is caused by Cas proteins mutating in order to avoid being inactivated by phage anti-CRISPRs. The rapid evolution of CRISPR-Cas systems makes their detection difficulty in metagenomic sequences of uncultured bacteria and archaea, because none of the existing known CRISPR-Cas systems in completely sequenced genomes is a close enough match. Although the new Cas proteins are structurally similar to known Cas proteins, the amino acid sequences have diverged to an extent that makes them difficult to detect even using the most sensitive sequence alignment methods (Remmert et al., 2012). While some Cas proteins such as Cas1 are easy to detect due to its very conserved sequence, other proteins, such as Cas8, are notoriously difficult to identify, owing to their strong sequence heterogeneity. Thus, even the most modern bioinformatics pipelines for annotation of genomic CRISPRCas loci have difficulties in detecting all *cas* genes comprising a complete CRISPR cassette.

According to comparative genomics studies of chromosomally encoded CRISPR-Cas systems (Garrett et al., 2011; Makarova et al., 2015; Shah et al., 2018; Vestergaard et al., 2014), these systems are carried on genomic cassettes, which are further divided into modules corresponding to the different functional stages of the immune response. Cassettes, as well as modules, are normally integral, meaning they have defined boundaries and are not intermixed with foreign genes. Thus, a typical bacterium may carry several Cas cassettes, and each cassette can be further divided into several operons, each corresponding to a functional module. Class 1 systems, in particular, have elaborated heteromultimeric interference complexes typically consisting of between four and eight genes. Knowing where the module starts and ends on the genome narrows down the possibilities and is an invaluable aid in annotating the *cas* genes that do not yield matches to any known Cas proteins.

Current bioinformatics pipelines for annotating *cas* genes treat each gene separately, while a cassette-aware pipeline could infer the identities of missing genes by simple exclusion (Alkhnbashi et al., 2014, 2016, 2020; Couvin et al., 2018; Crawley et al., 2018; Lange et al., 2013; Zhang and Ye, 2017).

In this article, we propose a new method to automatically define the boundaries of a CRISPR cassette. The proposed method takes into account the relation of a potential signature gene and genes that are contained in its neighboring region. Furthermore, the method labels the *cas* genes after the cassette boundaries have been defined, being also able to indicate genes that may belong to new putative types.

## 2 Materials and methods

This section introduces notation, definitions and problems addressed in this article. Afterwards, it describes our proposed method for cassette boundary detection and Cas type prediction in details.

### 2.1 Problem statement and notations

For a given genome, let $g_{1},\ldots ,g_{n}$ be all genes of the genome ordered by its genomic location (i.e. *g_i_* is located between $g_{i-1}$ and $g_{i+1}$ on the genome), and let $G=\{g_{i}|i\in [1:n]\}$ be the set of all genes in the genome. With $G_{c}$ we denote the set of all *cas* genes in this genome, and the set of all *cas* signature genes by $G_{s}$. $G_{u}=G\backslash G_{c}$ is the set of all *non-cas* genes.

We denote $S_{ij}\subseteq G$ as a set of consecutive genes $S_{ij}=\{g_{i},\ldots ,g_{j}\}$ and the set of all its consecutive subsets as $Sub(S_{\text{ij}})$. Note that $Sub(S_{\text{ij}})$ is not exponential in size as we considering only subsets that contain all genes in a genomic region. A consecutive subset *C* is called a *cassette* if it contains a sufficient number of *cas* genes and not too long stretches of *non-cas* genes. Formally, $C=S_{pq}$ is a cassette if

1.
$g_{p}\in G_{c}$ and $g_{q}\in G_{c}$ (first and last gene is a *cas* gene)
2.
gp−1∈Gc and gq+1∈Gc (the cassette is maximal)
3.
p−q+1≥3 (the cassette contains at least three genes)
4.
$\forall U\in Sub(S_{\text{pq}}):U\subseteq G_{u}\to |U|\leq 3$ (each consecutive subset of non-cas genes (called *gap*) is smaller than 3).

We call *g_p_* and *g_q_* lower bound and upper bound of the cassette, respectively. A cassette is often recognized by the presence of its signature gene, $g_{i}^{s}$. The set of all cassettes is notated as $G_{cs}$.

We formalize the problems addressed in this article as follows:

- *Cassette boundary detection*: in the first task, we aim at detecting the boundary for each cassette, given its signature *cas* gene. For such, we define a function $f_{c}(R,g_{i}^{s})$ that takes a potential region *R* and a signature gene $g_{i}^{s}\in R$ as its input and returns the boundaries of the maximal cassette $S_{pq}\in G_{cs}$ with $S_{pg}\subseteq R$ and $g_{i}^{s}\in S_{pg}$.
- *Cas type prediction*: in the second task, we want to determine the label for every *cas* gene. Formally, we define function $f_{l}:G_{c}\to L\cup \{N\}$, which maps a *cas* gene in $G_{c}$ to a label in L∪{N}, where *L* is the set of known Cas labels (such as Cas1, Cas2 etc.) and *N* is the label for unannotated *cas* genes.

### 2.2 Detection of cassette boundaries

In this section, we describe our proposed method for cassette boundary detection implementing the function *f_c_*. Our method is based on our assumption that the relation between a *cas* gene in a cassette and its signature gene is stronger than the relation for a gene that does not belong to that cassette. This assumption is motivated by the common understanding that signature genes $g^{s}\in G_{s}$ play an important role in defining the cassettes (Makarova *et al.*, 2015, 2020) and should be used as an anchor point in learning the cassette detection function *f_c_*. Furthermore, to simplify the problem of cassette detection, we define an auxiliary function $f(g_{j},g_{i}^{s})$ that is 1 (positive) if both genes are located in the same cassette and 0 (negative) otherwise. Thus, the first step of our method is to train a binary classification model for this auxiliary function *f*. We then use this trained model to detect cassette boundaries in a incremental manner as follows.

First, we slide over the genome. Whenever a signature gene $g_{i}^{s}$ is identified, a potential region, *R*, is defined for detecting the cassette boundary as $R=S_{i-k,i+k}$, where *k *>* *0 is large enough such that the full cassette is located inside this region. Next, the model is applied to predict the label for every tuple $(g_{j},g_{i}^{s}),\;g_{j}\in R$ starting from the genes located next to $g_{i}^{s}$ and extending the range in a stepwise manner. Finally, the boundaries *p*, *g* are predicted by Algorithm 1.

Theorem Let $R=S_{i-k,i+k}$ be the region around a signature gene $g_{i}^{s}$ and *S_pq_* be the associated cassette predicted by Algorithm 1. Then $S_{pq}=f_{c}(R,g_{i}^{s})$.

 

Proof Let $f_{c}(R,g_{i}^{s})=S_{s,t}\in G_{cs}$. First we note that $R\cap S_{pq}\neq \emptyset$ as both *R* and *S_pq_* contain $g_{i}^{s}$. To show equality, we proof by contradiction that there are no left-handed differences. The right-handed cases are analogous. Now lets assume that *s *<* p*. In this case, let *r* be maximal in s≤r<p≤i such that $g_{r}\in G_{c}$, which must exists as $g_{s}\in G_{c}$ by definition of a cassette. Then $U=\{g_{r+1},\ldots ,g_{p-1}\}\subseteq G_{u}$ by construction. As *S_st_* is a cassette and $g_{r}\in S_{st}\wedge g_{i}^{s}\in S_{st}$, we know that $f(g_{r},g_{i}^{s})=1$ and |U|≤3. Hence, *g_r_* would have been detected on the first loop of Algorithm 1 as it started from position *i *>* p* and must have considered position *p*, which is a contradiction.

For the other case let’s assume *p *<* s*. Note that *s* must have been visited in the first loop of Algorithm 1 as s≤i. Let be *g_r_* be a *cas* gene with p≤r<s≤i and *r* maximal. This must exist as $g_{p}\in G_{c}$ by the stop condition of the first loop in Algorithm 1. Let $U=\{g_{r+1},\ldots ,g_{s-1}\}$. By the first loop of Algorithm 1, we know $f(g_{r},g_{i}^{s})=1$ and |U|≤3. Thus, $S_{r,t}⊃S_{s,t}$ is a larger cassette, which is a contradiction to the maximality of $S_{s,t}$.

Finally, we get *s *=* p* and analogously *t *=* q*, which proofs our claim.

**Figure:**
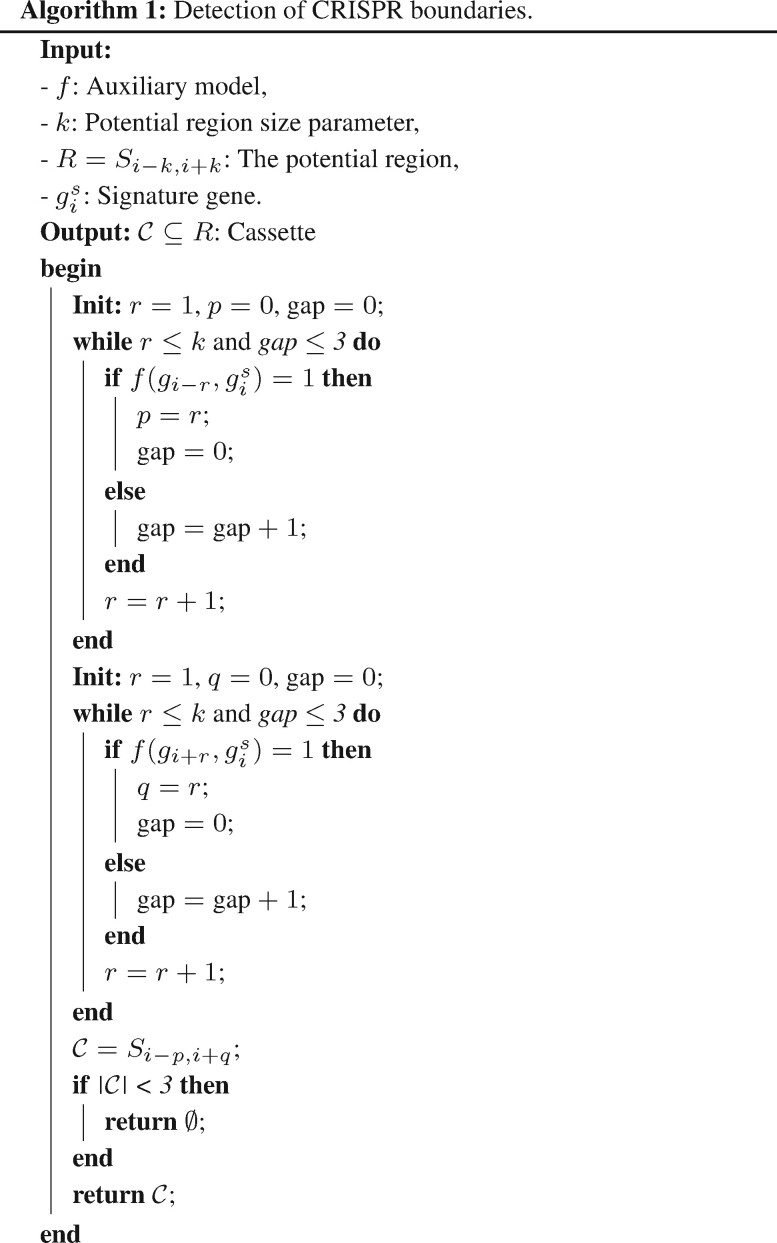


### 2.3 Classification of Cas proteins

Given the boundaries of a cassette, it is important to know the type of each *cas* gene in the cassette. A *cas* gene may belong to a set of predefined types or to a new type (i.e. previously undefined). To create a model able to identify the type of a *cas* gene, we train a multiclass classification model whose output indicates the probabilities of a given *cas* gene to belong to each Cas type. For such, we follow the procedure for word classification proposed in Shu et al. (2017), briefly described next:

1. We assume that the probability values of all examples *g_i_* that belong to each class *C_j_* are normally distributed and centered at $\mu (C_{j})=1$. To create the other half of the distribution, we mirror each of these probability values around $\mu (C_{j})$ (i.e. for each probability value $P(C_{j}|g_{i})$ associated to a training example *g_i_*, we create the artificial point $1+(1-P(C_{j}|g_{i}))$).
2. We estimate the standard deviation $\sigma (C_{j})$ using the obtained probabilities and the artificial mirrored values.
3. Finally, for each class *C_j_*, if the predicted probability for a test example *g_k_* is below the threshold $t(C_{j})=\max(0.5,\;1-\alpha \;\sigma (C_{j}))$, *g_k_* is considered as an outlier for *C_j_*. If the example is considered as an outlier for all classes, we label it as *N* (unnanotated). As suggested by Shu *et al.* (2017), we used *α* = 3. Otherwise, if the *cas* gene is not considered as an outlier, we assign to it the label with the highest probability.

In the original paper, Shu *et al.* (2017) used the training examples to estimate all thresholds $t(C_{j})$. However, in our study, we found out that this approach may yield overly optimistic estimations. To overcome this limitation, we used instead a validation set to estimate the thresholds.

### 2.4 Cassette modularization

Earlier studies (Garrett *et al.*, 2011; Shah et al., 2011; Vestergaard *et al.*, 2014) have found that Cas cassettes can be subdivided into discrete functional modules, with each module carrying out a separate function, and with its genes being spatially separate from other modules within the cassette. Annotating the constituent modules inside a cassette can reveal important information in terms of the functional organization of the CRISPR-Cas system. Typically, a cassette is composed by three types of modules: adaptation, processing and interference. The processing module typically consists of a single *cas* gene, which is located either close by the interference module or far away from the region defined by the cassette boundaries. For these reasons, we take only the adaptation and interference modules into account. The adaptation module contains genes that are the most conserved across different genomes, being easy to detect. Therefore, in the first step of our method, we want to detect the adaptation module by searching for a sub-region containing Cas1, Cas2 and/or Cas4. Next, the sub-regions which are adjacent to the adaptation module will be considered as the interference modules.

In CRISPR-Cas field, a cassette can have one or more interference modules. Based on the number of interference modules, we define cassettes with a single interference module as *single cassettes* and cassettes with more than one interference modules as *multi-module cassettes*. Note that the interference modules in a multi-module cassette might be overlapped or separated.

## 3 Empirical evaluation

### 3.1 Data collection and preprocessing

We collect CRISPR data publicly available from Makarova *et al.* (2015, 2020). Our dataset has 52730 Cas proteins, with 7793 CRISPR cassettes distributed into 22 different subtypes (see Supplementary Table S1). We download the genomes from the NCBI database and extract the Cas protein sequences by applying the Prodigal tool v2.6.3 (Hyatt et al., 2010) on the respective gene sequences. For each CRISPR cassette, we identify its signature gene $g_{i}^{s}$, the most important gene to define the cassette of interest (Makarova *et al.*, 2015, 2020). Next, we extract *k* genes downstream and *k* genes upstream to *g_s_*. Usually, the length of a CRISPR cassette ranges from 3 to 15 genes (Makarova *et al.*, 2015, 2020). Thus, we set *k *=* *50, which safely includes the full cassette in the extracted region.

To define the features for each gene, we use three different types of features, described as follows:

1. *General HMM features*: we collect all available Hidden Markov Models (HMM) from the following public databases: TIGRFAM (Haft, 2003), Pfam (Bateman, 2004), COG (Tatusov, 2000) and CDD (Marchler-Bauer et al., 2011), totalizing 38847 HMMs. For each protein sequence, the features are defined as the bitscores generated by each HMM. We reduce the number of features to 500 using the Truncated Singular Value Decomposition (Manning et al., 2009), with 60% of the original data variance preserved.
2. *Protein properties features*: we calculate 12 features related to the properties of each extracted protein, such as: molecular weight, length, isoelectric point, number of negatively charged residues, number of positively charged residues, extinction coefficient (with and without cysteine), instability index, hydrophobicity and secondary structure properties (fraction of turn, sheet and helix).
3. *Specific HMM features*: we build 623 HMM models for the different Cas protein models based on the core and signature genes from the dataset used (Makarova *et al.*, 2015, 2020). Since these HMM models are more specific to the CRISPR domain, we believe that they may be better suited for the task of identifying potentially new Cas types.

We create a dataset of 7793 cassettes, out of which 7687 are single cassettes, such as those illustrated inFigure 1. Each one of the remaining 106 cassettes, which are multi-module cassettes, can be decomposed into two or three single cassettes whose signatures are close in the genome. We divide these 106 cassettes into 2 subgroups: (i) the *Separated set*, which contains 74 multi-module cassettes that can be broken up into 145 single cassettes that do not overlap (e.g. see Fig. 2a); and (ii) the *Overlapped set*, which contains 32 multi-module cassettes that can be broken up into 70 single cassettes that present some degree of overlap (e.g. see Fig. 2b).

**Fig. 1.**
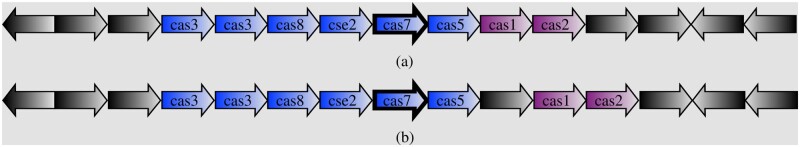
Examples of the structure of CRISPR cassettes: (**a**) single CRISPR cassette; and (**b**) single CRISPR cassette with a gap. The signature genes are in bold. Blue arrows are interference genes while purple arrows are adaptation genes

**Fig. 2.**
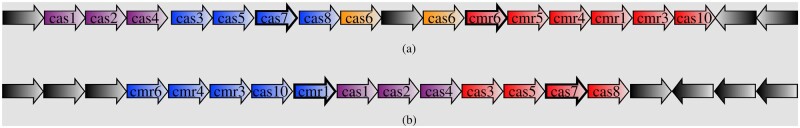
Examples of the structure of multi-module CRISPR cassettes: (**a**) multi-module cassette without overlap; and (**b**) multi-module cassette with overlap. The signature genes are in bold. The blue and red arrows are interference genes, yellow arrows are processing genes and purple arrows are adaptation genes

### 3.2 Machine learning algorithms

Our method for cassette boundary detection requires a binary classification model, whereas the Cas type prediction demands a multiclass classification model. In our experiments, we use two algorithms to train them which, in addition to be known for their good performance in several tasks, have different learning biases:

- *Extremely Randomized Trees (ERT)* (Geurts et al., 2006), which is a classifier that integrates multiple decision trees in an ensemble. To define the splits for each tree, this method selects, at each step, a random subset of *v* features and a subset of *v* thresholds (one for each feature). Afterwards, the feature that contains the best randomly chosen threshold according to the quality criterion is selected. After training, the class predicted for unseen examples is defined by the majority vote of all trees.
- *Deep Neural Networks (DNNs)* (Goodfellow et al., 2016), which are neural networks with a large number of layers whose neurons’ total input is a dot product between a numeric vector input and the neuron’s synaptic weights followed by the application of a non-linear activation function. By using the first layers to extract relevant features, DNNs can learn highly complex functions. DNNs are usually computationally expensive to train. However, with the recent advances in the computer processing power, they have obtained the best predictive performance in a wide range of applications (Liu et al., 2017).

### 3.3 Experimental setup

Two experiments are carried out to evaluate the predictive performance of the proposed method. The first assesses the ability of our method to detect cassette boundaries. For such, we use 10-fold cross-validation for the dataset with 7687 single cassettes, separating one of the training folds for validation, and hold-out for the dataset with 106 multi-module cassettes. The second experiment evaluates how well the proposed method classifies Cas proteins. In this experiment, we employ hold-out for a dataset with 52730 Cas proteins.

*Cross-validation*. We split the data into 10-folds. Before training, we undersample the majority (negative) class, to mitigate the negative effect of data imbalance on the model training. We repeat the experiment 10 times and report the average and standard deviation of the performance over the 10 × 10 runs.

*Hold-out*. For the Cas type classification, we leave 20% of the data out for testing and the remaining for training (80%) and a fifth of the training set, for validation. To evaluate the performance for undefined *cas* genes, we leave in turn one and three Cas types out of the training and validation set to simulate undefined Cas types scenarios. We repeat this procedure to ensure that every Cas type is left out once. We run the experiment 10 times. Regarding the boundaries detection for multi-module cassettes, we use the 7687 single cassettes as a training and validation set and the 106 multi-module cassettes as the test set.

*Model selection*. To tune the hyperparameters of each learning algorithm, we employ the grid search with 32 different hyperparameter combinations. For ERT, we tune the number of trees in {50,100,150,200}, the number of features randomly selected for each split in $\left\{\sqrt{m},\;{\text{log}}_{2}m\right\}$ and the minimum number of examples to be at a leaf node in {1,4,7,10}. For DNNs, we use two hidden layers and vary their numbers of neurons in {25,50,75,100}, the Adam optimizer (Kingma and Ba, 2015) and consider the learning rate values in {0.01,0.001}. Concerning maximum gaps, we consider values between 0 and 3.

*Evaluation metrics*. For the evaluation of cassette boundaries detection, we use the following measures: where $C^{t}$ and $C^{p}$ are the true and the predicted cassette, respectively. This measure lies in the interval [0,1] where 1 indicates a perfect match. where *p^t^* (resp. *p^p^*) and *q^t^* (resp. *q^p^*) refer to the index of the first and the last gene of the true (resp. predicted) cassette, respectively. This measure lies in the interval [0,∞) where 0 indicates a perfect match, i.e. the boundaries of the predicted cassette are in perfect agreement with true cassette. Intuitively, CL denotes the average boundary deviation for the left and right end together.

- The Jaccard Similarity (JS), which is a popular measure for comparing different sets and is defined as:
-
$\text{JS}(C^{t},C^{p})=\frac{\left|C^{t}\cap C^{p}\right|}{\left|C^{t}\cup C^{p}\right|},$

- The Cassette Loss (CL), which is an adaptation of the mean absolute error, a popular measure for the evaluation of regression tasks. CL quantifies the gene-wise mean absolute error and is defined as:
-
$\text{CL}(C^{t},C^{p})=\frac{\left|p^{t}-p^{p}|+|q^{t}-q^{p}\right|}{2},$

For the evaluation of the Cas protein classification, we use the *F*-score with macro-averaging. Given a binary classification task where we have a specific class of interest (positive class), the classical *F*-score is defined as:

$$F-\text{score}=\frac{2\text{TP}}{2\text{TP}+\text{FP}+\text{FN}}$$

where TP, FP and FN correspond to the number of true positives, false positives and false negatives, respectively. For the multiclass scenario, the macro-averaging consists of calculating the *F*-score for each individual class and reporting the average *F*-score as the global performance measure. The main advantage of macro-averaging is that it treats all classes with the same weight, independently of the number of examples that they contain (Sokolova and Lapalme, 2009).

## 4 Results and discussion

In this section, we report and analyze the results obtained from our experiments.

### 4.1 Detection of cassette boundaries

**Fig. 3.**
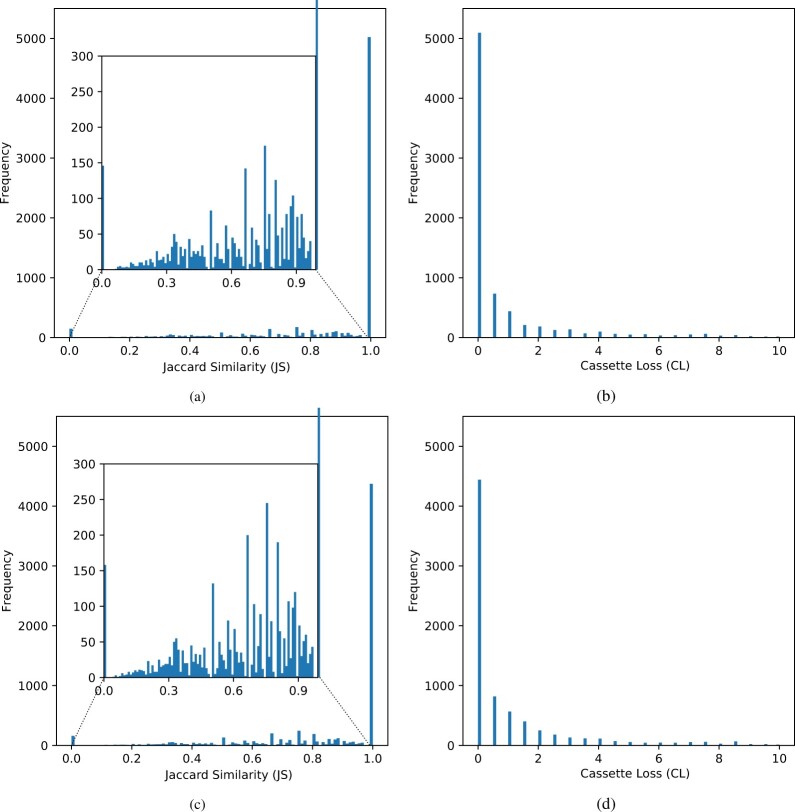
Histogram containing 100 equally sized bins of the Jaccard Similarity and Loss for single cassette prediction using ERT (**a, b**) and DNN (**c, d**). The inner figures are the zoom of the corresponding outer ones without considering the most dominant bin

**Fig. 4.**
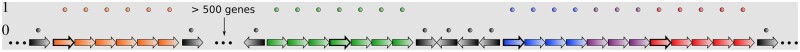
Examples of our method’s cassette prediction for the organism Thermotoga sp. RQ2. Specifically, it found two cassettes composed by single interference modules, represented by the orange and green arrows, and a multi-module cassette with two interference modules (blue and red arrows) and an adaptation module (purple arrows). See Figure S3 for more details

We report the histogram of JS and CL values for single cassette prediction in Figure 3, using only the general HMM features, which were our best results. For the histograms of other types of features, please check our Supplementary Material (Supplementary Figs S1 and S2). From Figures 3a and c, it can be noticed that most of the JS values are 1.0 and CL values are 0.0, indicating that our model is able to correctly predict most of the cassettes. In addition, in Table 1, we show the average JS and CL values that we obtained for both single and multi-module cassettes. When comparing our results to those achieved by CRISPRCasFinder (Couvin *et al.*, 2018), the closest tool to our method, it is possible to note that we achieved around 16% of JS improvement in the best case for single cassettes. In particular, our tool would predict cassette boundaries correctly with a precision of roughly one position, whereas CRISPRCasFinder would be roughly five positions away on average. Regarding multi-module cassettes, we obtained JS values above 0.70, while CRISPRCasFinder achieved extremely low JS values which are less than 0.15 in both separated and overlapped cases. It confirms the superiority of our method over CRISPRCasFinder in the detection of cassette boundaries. Besides, to illustrate the capability of our method in this scenario, we present in Figure 4 an example of cassette prediction for the organism Thermotoga sp. RQ2.

**Table 1.** Performance of our method and CRISPRcasFinder for the identification of single and multi-module cassettes in terms of JS and CL

Method	Single cassettes		Multi-module cassettes			
			Separated set		Overlapped set	
	JS	CL	JS	CL	JS	CL
ERT	0.86 ± 0.01	1.09 ± 0.12	0.79	1.10	0.72	1.93
DNN	0.83 ± 0.01	1.39 ± 0.20	0.74	1.77	0.73	2.21
CRISPRcasFinder	0.70	4.87	0.13	30.52	0.10	19.88

*Note*: For multi-module cassettes, the prediction quality for boundary detection drastically drops for CRISPRcasFinder, whereas our tool has similar performance to the single cassette case.

### 4.2 Classification of Cas proteins

In Figure 5, the average *F*-scores for Cas type prediction of our method using a combination of specific HMMs and gene properties features are shown. For details of the performance of the models using different types of features, please see our Supplementary Material (Supplementary Figs S3–S11).

**Fig. 5.**
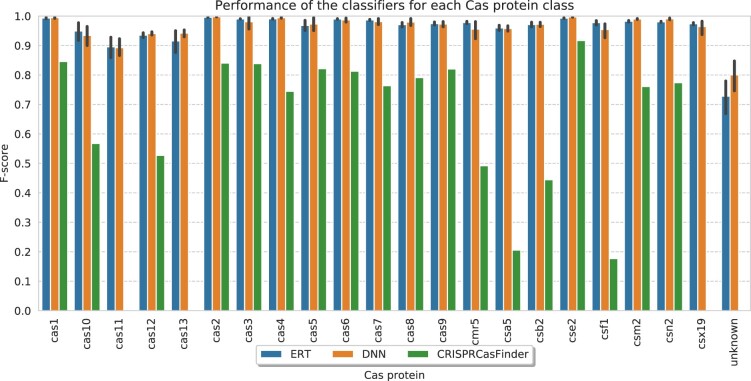
Comparison of Cas type prediction *F*-scores between our models (using a combination of the specific HMM and protein properties features) and CRISPRCasFinder. For a comparison between the runtime of Casboundary and CRISPRCasFinder, see Supplementary Table S3

Overall, our method achieved high predictive performances for all Cas types using both ML models. More precisely, for the known Cas types predictions most values are equal to or higher than 0.9. Regarding the prediction of unknown Cas types, ERT and DNN achieved average *F*-scores of 0.73 and 0.80, respectively. Although the results for unknown Cas types are relatively lower than those of known Cas types, this reduction is expected, given the difficulty of the task for detecting new classes caused by the balancing between the high *F*-scores for known classes and the ability to potentially point out new genes. The high predictive performance of our models shows their potential for the classification of Cas types for genes in general and for un-predefined *cas* genes observed in many cassettes in particular.

### 4.3 Prediction of potentially new Cas proteins

In this task, we use our method to investigate the problem of predicting (potentially) new Cas proteins, which is a typical scenario for the analysis of novel cassettes. For such we integrated into our method the best ML models that we obtained in the previous section. They are able not only to integrate the knowledge extracted from multiple HMM models and protein properties, but also to generalize the relations among those features.

**Fig. 6.**
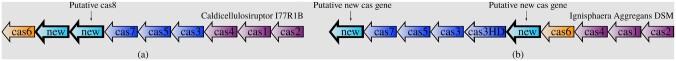
Examples of the application of our method for the identification of potentially new Cas proteins, which are marked in bold. In (**a**), our method predicted two proteins as ‘new’, where one of them has some similarity with Cas8 proteins and may be a new subfamily of Cas8. In (**b**), our method predicted two proteins as ‘new’, which do not have any similarity to other known Cas proteins and may indicate two new genes

First, given the cassette boundaries for a genome, we applied our classification methods to label each protein contained in it. Then, we analyzed the proteins that were labeled as ‘unknown’, by performing a clustering search against our database. In Figure 6a, our method labeled two proteins as potentially new. One of them presented a good degree of similarity with a few Cas8 proteins (see our Supplementary Material, Supplementary Figs S10–S12). Since this family is very diverse, this result suggests that it may belong to a new Cas8 subfamily and we labeled the respective gene as ‘putative cas8’. In Figure 6b, our method labeled two genes as potentially new. We did not find any convincing resemblance with the proteins we had in our database. Thus, we believe that such proteins may represent new protein families and we label the respective genes as ‘putative new *cas* gene’. See the Supplementary Material for more details.

### 4.4 Occurrence of exchangeable modules

CRISPR cassettes are multi-module structures which are made up of several functional modules each responsible for their own stage of the immune response (Vestergaard *et al.*, 2014), including adaptation, processing and interference, in addition of optional accessory modules. The genes comprising each module within a cassette are separated from each other into distinct operons, such that the modules themselves are integral (Shah *et al.*, 2011). Such a structure enables differential regulation of the expression of the different immune stages, but also enables independent horizontal transfer of a module within a cassette without affecting the functionality of the rest of the immune response. There have been previous reports of CRISPR cassettes from related organisms having undergone such shuffling of modules (Garrett *et al.*, 2011), although no systematic survey has been made. The capability of our method to define the edges of both Cas modules and cassettes was employed on a database of bacterial and archaeal genomes (Section 3.1) and the identities of the detected modules were compared in order to gauge the extent of modular exchange in natural CRISPR-Cas systems.

All cassettes consisting of no more than a single adaptation module and a single interference module were included in the analysis. Adaptation modules from different cassettes were aligned against each other in order to determine their similarity degree (see the Supplementary Material). The subtype of each cassette was determined by looking at the interference module. Finally, for each adaptation module, the subtype of its closest match from a different cassette was recorded in Supplementary Table S2.

Subtypes with a high diagonal percentage close to 100 almost never share their adaptation module with other subtypes of interference modules. I-E and I-F are a good examples of such subtypes, and this observation is consistent with that fact that the adaptation and inferencence stages are coupled in systems of these subtypes, with Cas3 being involved in both stages (Vorontsova et al., 2015; Westra et al., 2012). On the contrary, and consistent with earlier reports (Garrett *et al.*, 2011; Vestergaard *et al.*, 2014), subtypes I-A, I-B, I-D frequently engage in modular exchange, probably because the adaptation and interference stages are independent in these subtypes (Plagens et al., 2012). Besides, most Type III systems have been known for long to piggyback on adaptation and processing machineries of co-occurring Type I systems (Haft et al., 2005; Hale et al., 2009; Makarova et al., 2011) because they have no such modules of their own, explaining their particularly low diagonal percentages. The extremely low diagonal percentage (37) found for subtype I-U suggests very frequent modular exchange comparable to Type III systems. This result indicates that the subtype co-functions with other CRISPR-Cas systems belonging to subtypes as I-A, I-C and Type III. This subtype may not have specific adaptation system of its own, like Type III systems. Given that very little experimental data exists on subtype I-U systems, these observations still need confirmation.

### 4.5 Automated annotation of Cas Cassettes and modules

We made our method available as an open source tool in GitHub. It was implemented in Python and is based on the method that integrates our best ML models. Casboundary accepts a complete or partial genome sequence as input, identifies the potential signature genes by using Cas-specific HMM models (Makarova *et al.*, 2020) (see Section 3.1), and provides a full identification of the CRISPR cassettes. Next, it labels the genes of the cassette and, as a post-processing step, it can also perform the decomposition of the identified cassette into modules.

Casboundary can be easily integrated with CRISPRcasIdentifier (Padilha et al., 2020), a recent tool for the classification of CRISPR cassettes. Casboundary outputs a set of Fasta files containing the identified cassettes, which can be given as input to CRISPRcasIdentifier.cAs a next step, CRISPRcasIdentifier can classify each cassette into its respective subtype and also predict potentially missing proteins in it. By integrating these tools, the users have a complete CRISPR detection and classification pipeline.

## 5 Conclusion

In this article, we introduce the first method for automated cassette boundary detection, Cas protein annotation and classification. We apply our method on the datasets from Makarova *et al.* (2015, 2020), which comprise single and multi-module cassettes. Additionally, we also present two real study cases, where we analyze the occurrence of exchangeable models and the prediction of potentially new Cas protein classes.

With respect to boundary detection, the approach followed by our method combines the information available for different genes and a potential signature gene of interest. In our experiments, the method obtains promising predictive performance results as measured by the JS and CL. For single cassettes, we obtain an average JS of 0.86 and CL below 1.09 with the best ML model. For composite cassettes, such a model reaches average JS (resp. CL) values of 0.79 (resp. 1.10) and 0.72 (resp. 1.93) for separated and overlapped cassettes, respectively.

Concerning the Cas protein classification, our method is not only able to assign the Cas type labels for known Cas proteins but also to label a Cas protein as a potentially new type. In our experiments, where we simulate the occurrence of new Cas types by leaving out either 1 or 3 subtypes, our models achieve *F*-scores above 0.9 for known cas types. Besides, we perform a real study case where our method is able to suggest new putative *cas* genes. Moreover, we conduct another study case to analyze the occurrence of exchangeable models in CRISPR-Cas systems. Our analysis presents evidence of the exchange of adaptation and interference modules in different archea and bacteria CRISPR-Cas systems.

Finally, our method is available as an open source tool in GitHub. At each run, it loads our best ML models and allows the user to apply all the developed methods in an easy and pragmatic way to new CRISPR cassettes.

## Funding

This work was funded by the Deutsche Forschungsgemeinschaft (DFG, German Research Foundation) under Germany’s Excellence Strategy—EXC-2189—Project ID: 390939984, BA 2168/11-1 SPP 1738 and BA2168/11-2 SPP 1738, BA 2168/3-3, and GRK 2344/1 2017 MeInBio, and the São Paulo Research Foundation (FAPESP) [2013/07375-0 and 2019/21300-9].

**Conflict of Interest:** None declared.

## Supplementary Material

btaa984_Supplementary_Data
