# Supplementary material for: Casboundary: automated definition of integral Cas cassettes
Source: Bioinformatics. 2020 Dec 6;37(10):1352–9. doi: 10.1093/bioinformatics/btaa984 (PMC8208735; doi:10.1093/bioinformatics/btaa984)
Supplement: btaa984_Supplementary_Data [file btaa984_supplementary_data.pdf]

Casboundary: Automated Definition of Integral Cas Cassettes
Supplementary Material

Victor A. Padilha, Omer S. Alkhnbashi, Van Dinh Tran, Shiraz A. Shah, André C. P. L. F. Carvalho and Rolf Backofen

In Table S1, we show the distribution of CRISPR cassettes into the 22 different subtypes contained in the collected dataset.

In Figures S1 and S2 we present the remaining results for the cassette boundary detection, using a combination of the General HMM features with the protein properties features and using only the protein properties features, respectively.

In Figures S4–S12 we present the additional results for Cas type classification.

In Figures S13–S15 we show the results for the study case on the identification of potentially new Cas proteins.

Finally, in Table S2, we show the results of the occurrence of exchangeable modules.

Table S1: Number of cassettes for each CRISPR subtype in the collected dataset.

CRISPR Subtype	# of cassettes	CRISPR Subtype	# of cassettes
I-A	146	III-B	377
I-B	976	III-C	72
I-C	855	III-D	257
I-D	135	III-E	1
I-E	2122	IV-A	105
I-F	698	V-A	38
I-U	169	V-F	27
II-A	722	VI-A	6
II-B	60	VI-B	53
II-C	563	VI-C	5
III	1	VI-D	1
III-A	518		

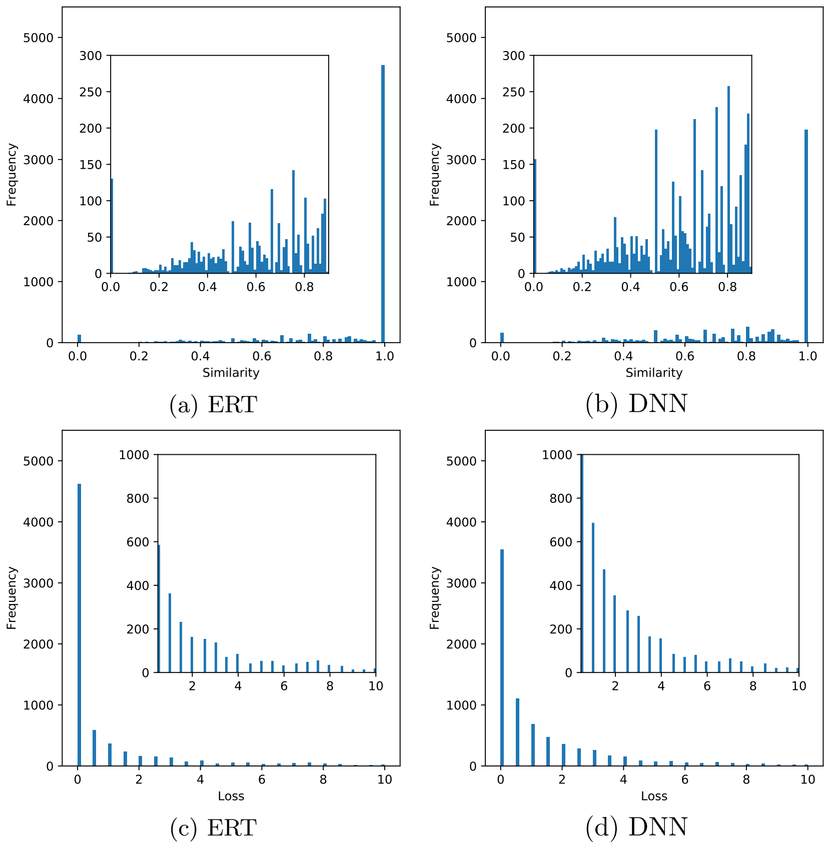

(a) ERT (b) DNN

(c) ERT (d) DNN

Figure S1: Histogram of the JS and CL for single cassettes using general HMM and protein properties features.

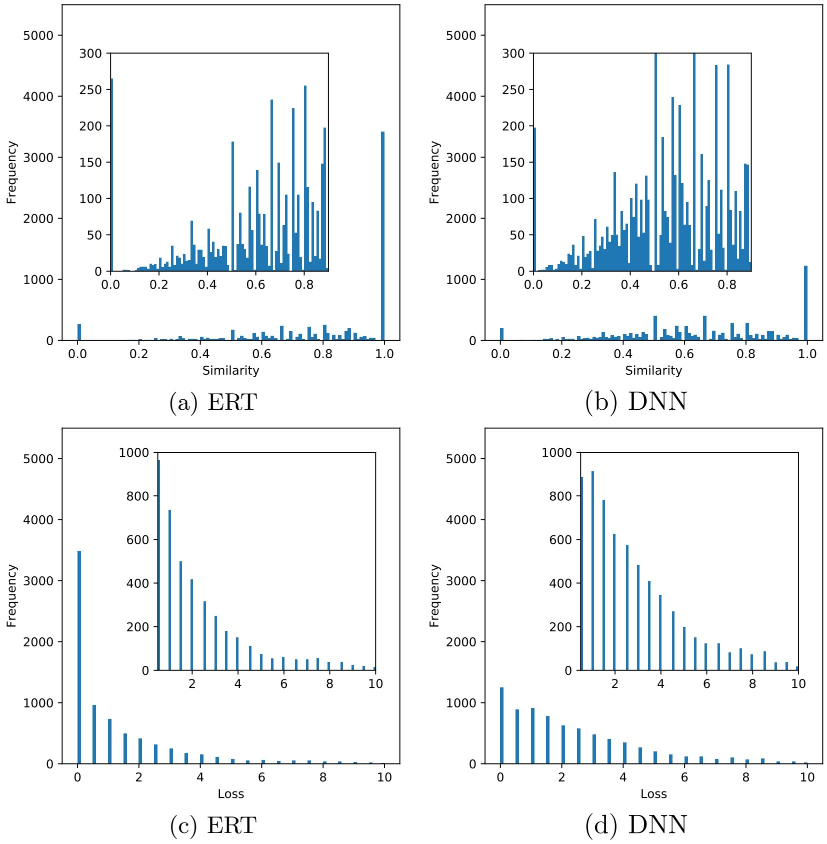

Figure S2: Histogram of the JS and CL for single cassettes using protein properties features.

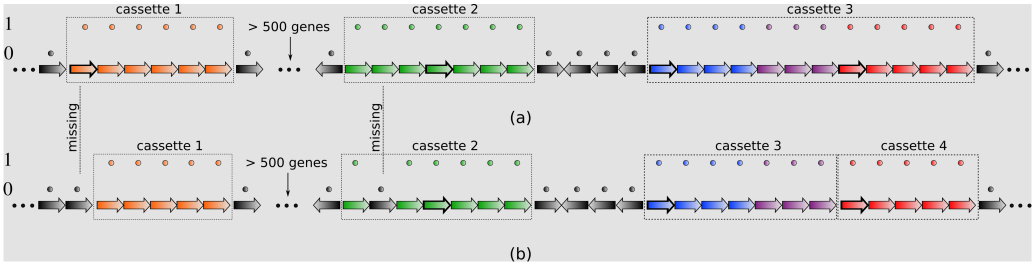

Figure S3: Comparison between (a) our method's and (b) CRISPRCasFinder's cassette prediction for the organism Thermotoga sp. RQ2. The genome has two single cassettes and one multi-module cassette. Our tool can easily handle those cassettes by identifying them as it should be in nature. In contrast, CRISPRCasFinder struggles to report such cases. For example, in cassette 1 (orange) and cassette 2 (green), one gene is missing. Moreover, cassette 3 and 4 must be one cassette containing three different modules (two interference modules and a single adaptation module). However, CRISPRCasFinder splits it into two different cassettes, which is different to what is expected in nature.

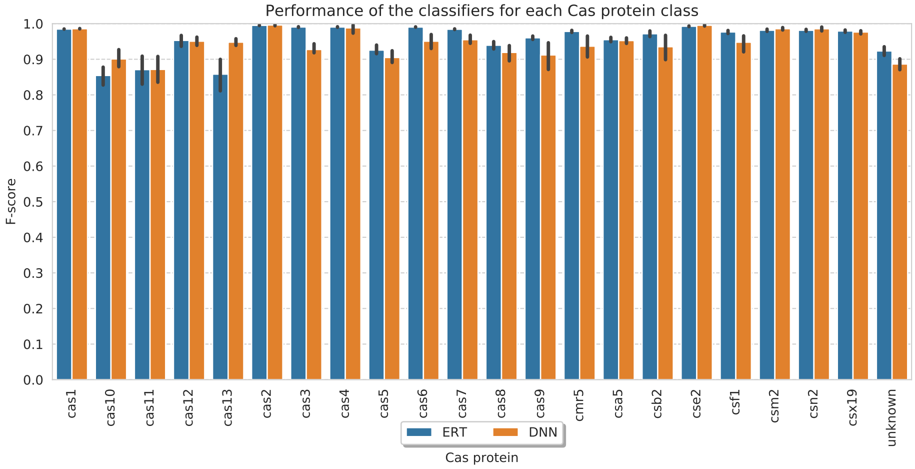

Figure S4: Cas type prediction F-scores with 3 cas types left out, using a combination of the specific HMM and protein properties features.

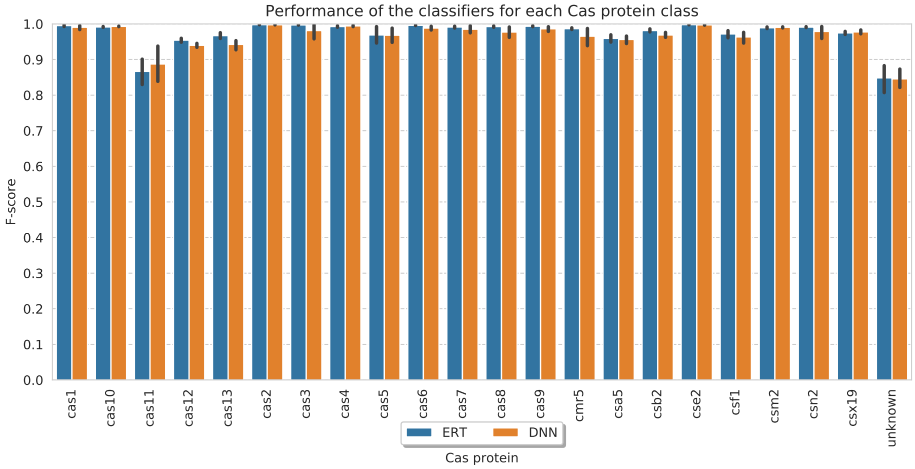

Figure S5: Cas type prediction F-scores with 1 cas type left out, using the specific HMM features.

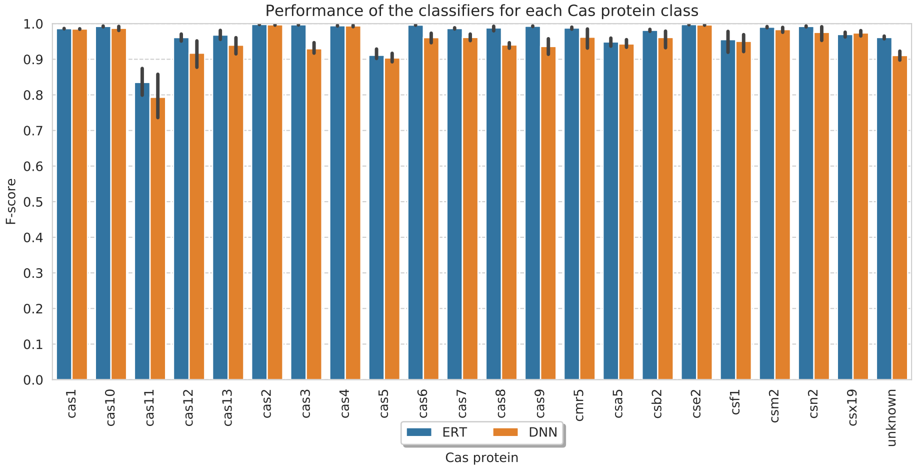

Figure S6: Cas type prediction F-scores with 3 cas types left out, using the specific HMM features.

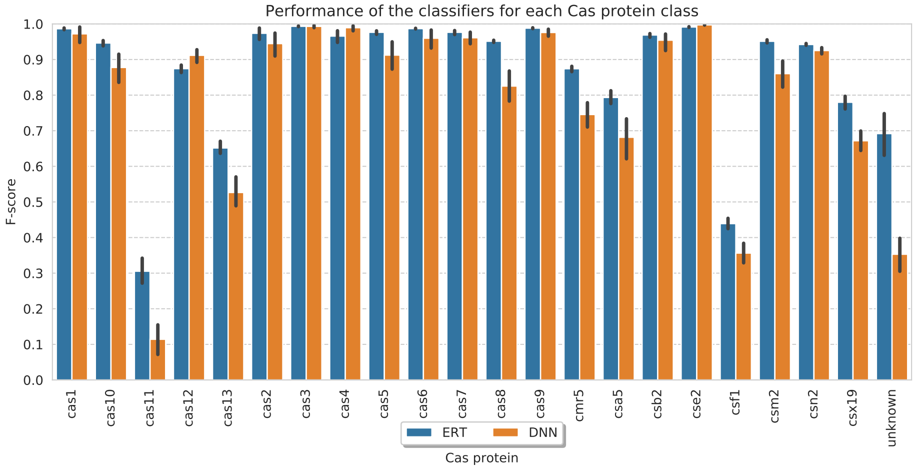

Figure S7: Cas type prediction F-scores with 1 cas type left out, using the general HMM features.

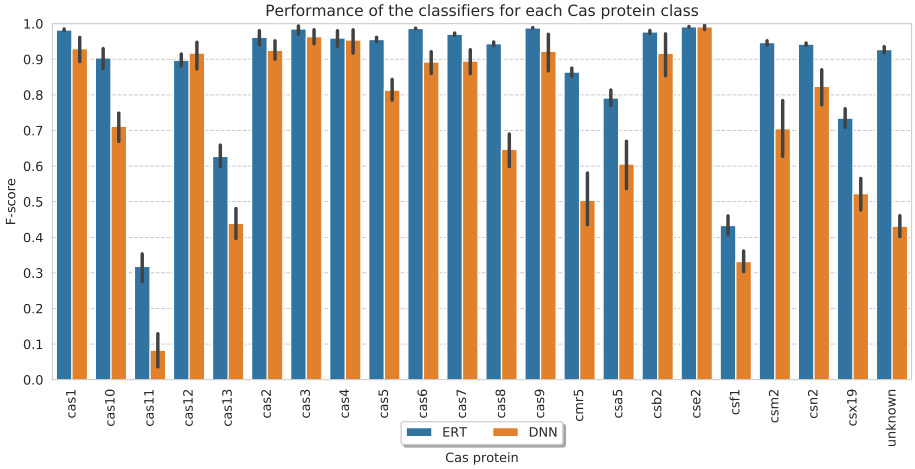

Figure S8: Cas type prediction F-scores with 3 cas types left out, using the general HMM features.

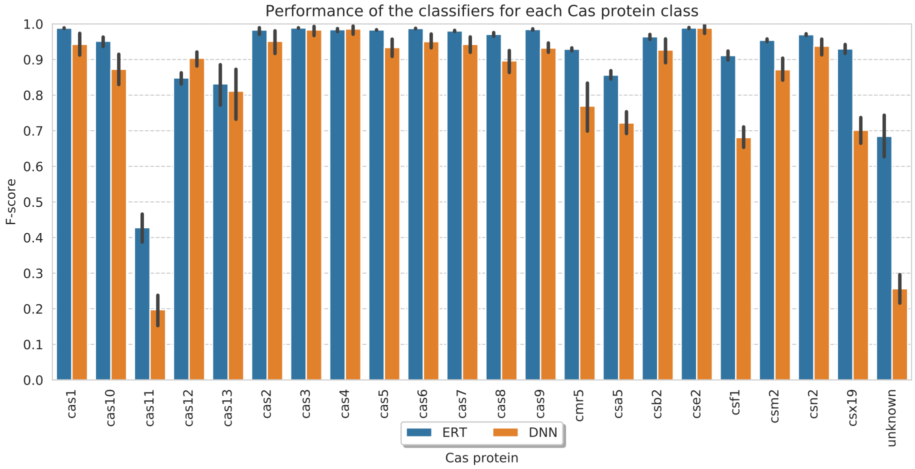

Figure S9: Cas type prediction F-scores with 1 cas type left out, using a combination of the general HMM and protein properties features.

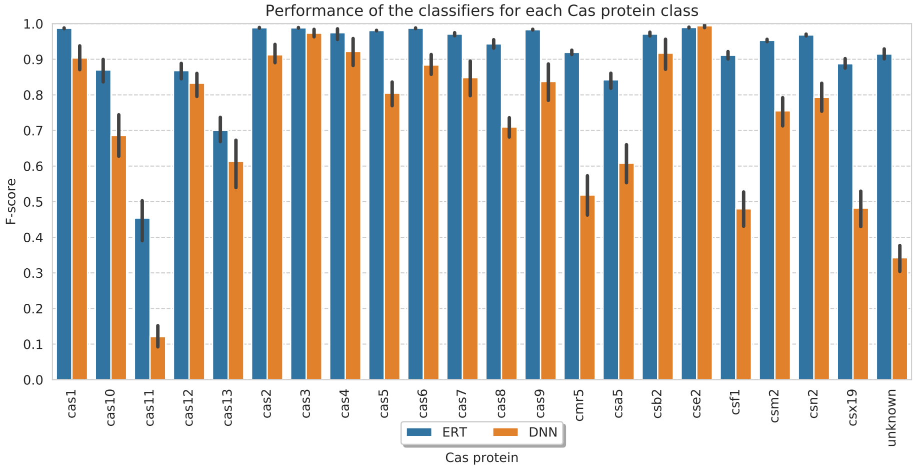

Figure S10: Cas type prediction F-scores with 3 cas types left out, using a combination of the general HMM and protein properties features.

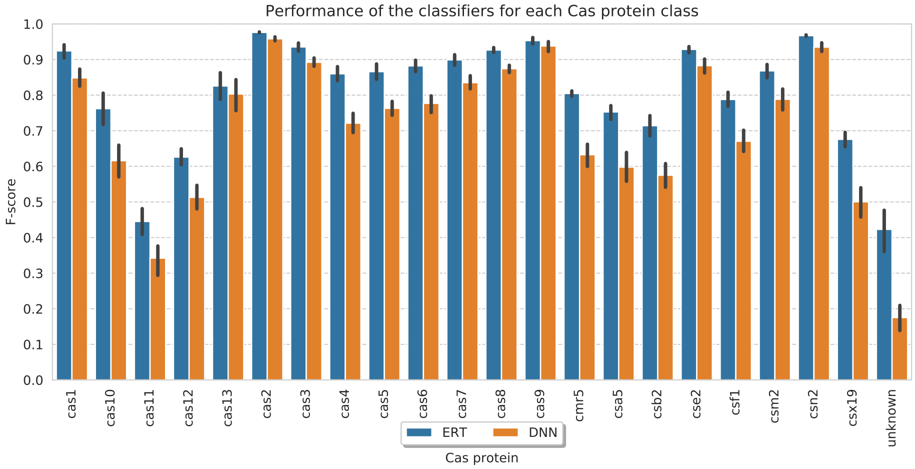

Figure S11: Cas type prediction F-scores with 1 cas type left out, using the protein properties features.

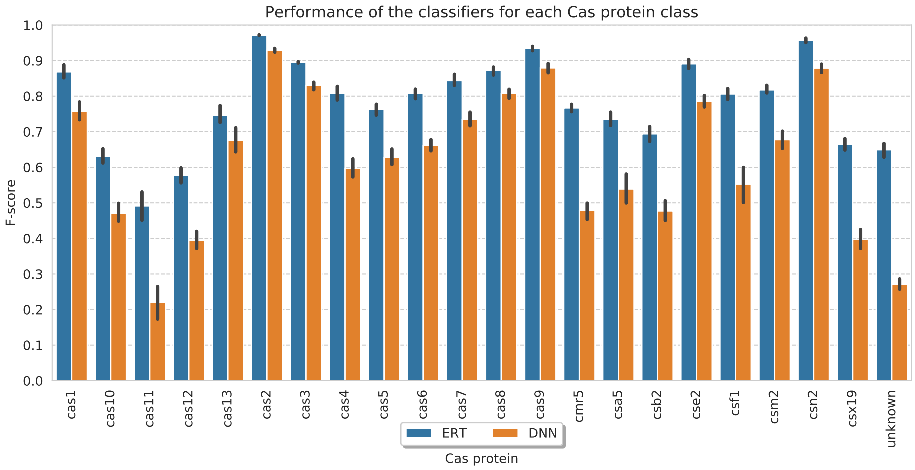

Figure S12: Cas type prediction F-scores with 3 cas types left out, using the protein properties features.

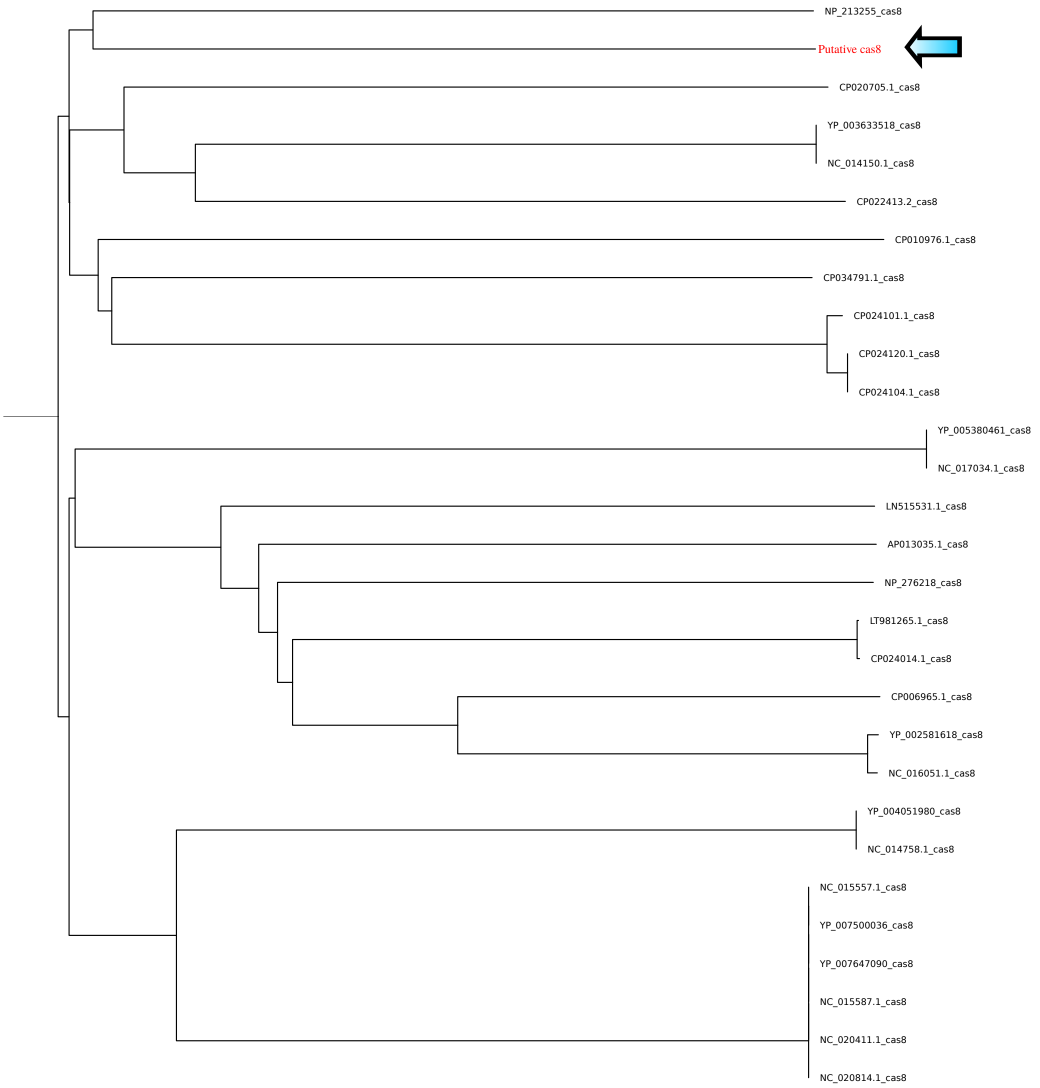

Figure S13: Phylotree of Cas8 and putative cas8. The tree is generated based on the Neighbour-joining method. Here we showed the distance between the closest 29 proteins from Cas8-family along with the putative cas8 protein.

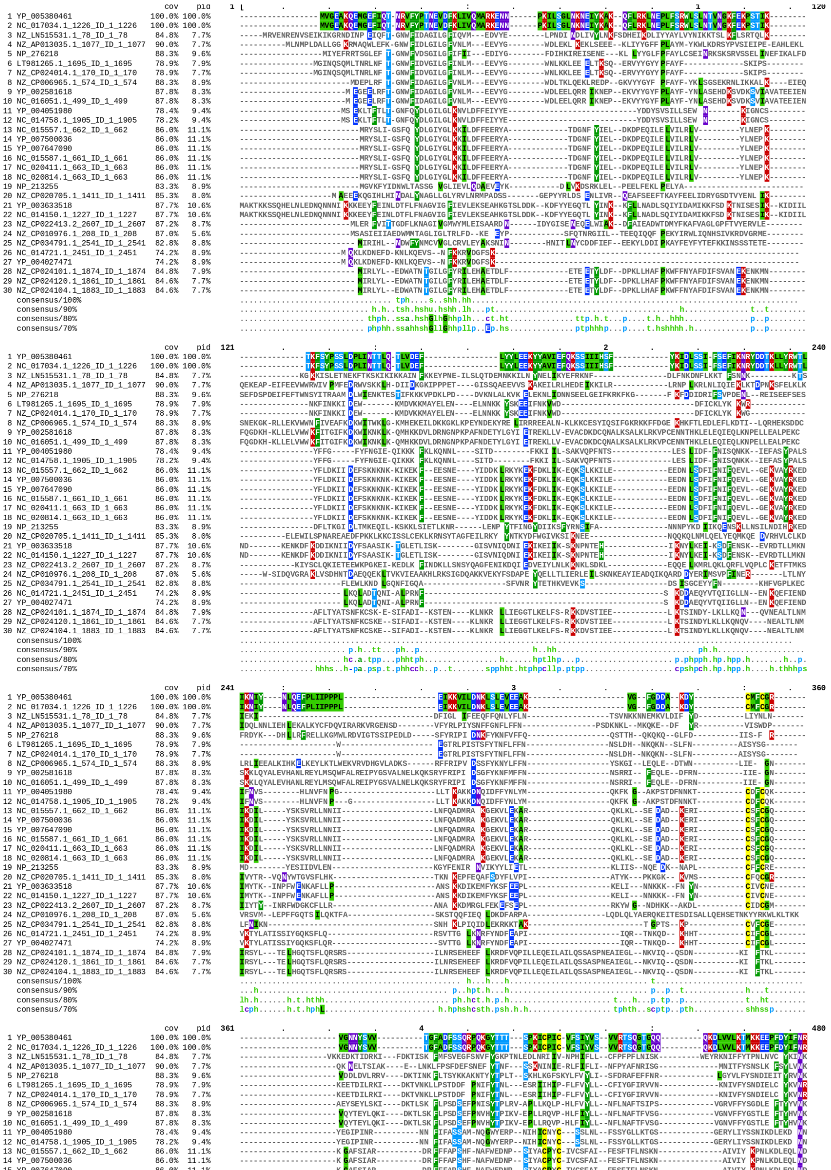

Figure S14: Multiple Sequence Alignment of Cas8 proteins and the putative Cas8 proteins. The alignment obtained from MUSCLE alignments of 29 Cas8 family and the putative Cas8. The conserved regions are shown on the bottom of the alignment.

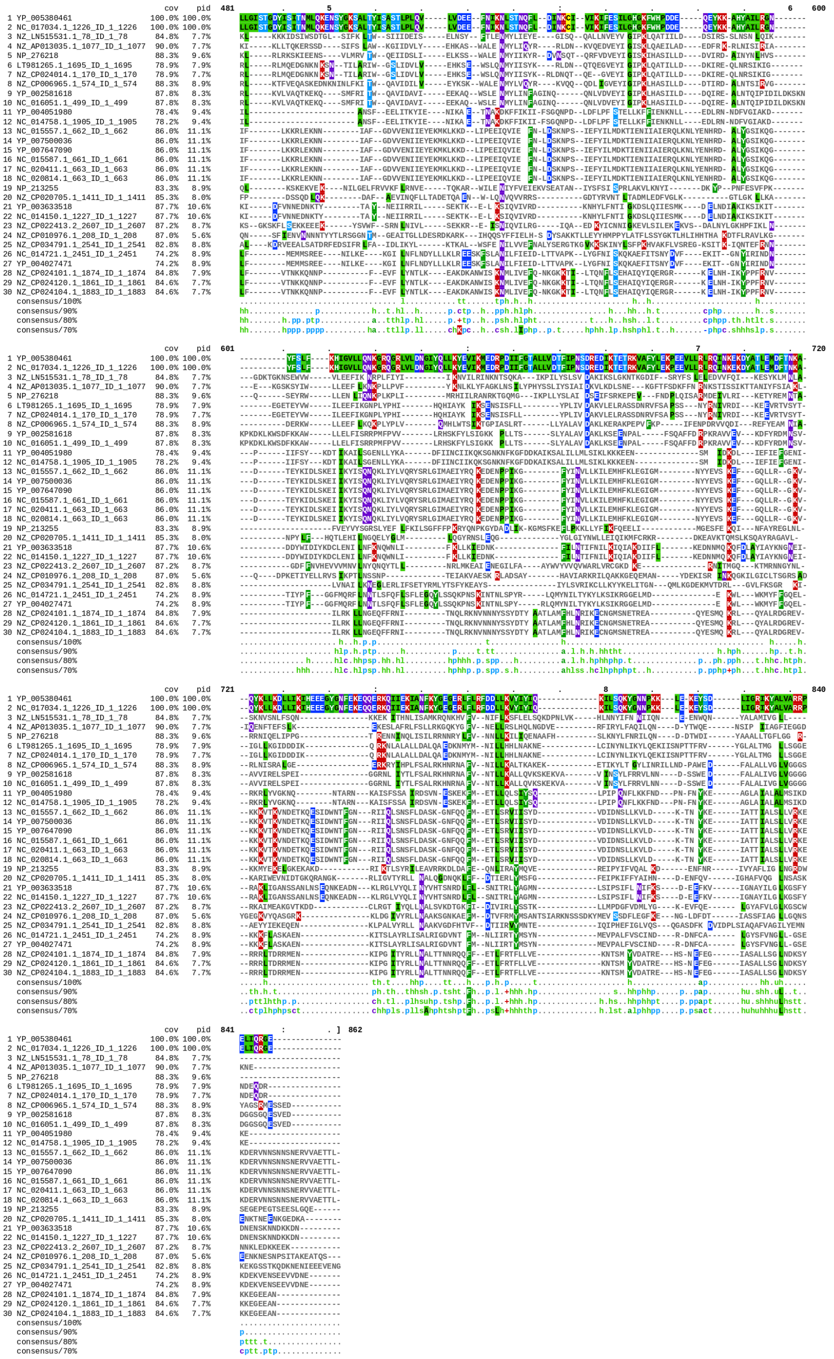

Figure S15: Multiple Sequence Alignment of Cas8 proteins and the putative Cas8 proteins. The alignment obtained from MUSCLE alignments of 29 Cas8 family and the putative Cas8. The conserved regions are shown on the bottom of the alignment.

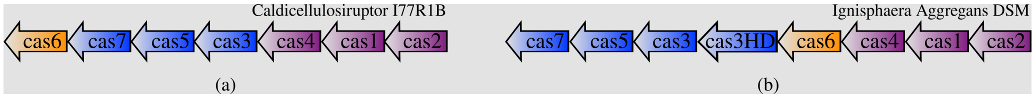

Figure S16: CRISPRCasFinder's results for the examples presented in Figure 6. This tool was able to detect only the known cas genes.

Table S2: Percentage of closest matches for Cas1 under each subtype. The analysis was carried out using the k-nearest neighbors approach with $k = 5$.

	I-A	I-B	I-C	I-D	I-E	I-F	I-U	II-A	II-B	II-C	III-A	III-B	III-C	III-D	III-E	IV-A	V-A	VI-A
I-A	66.59	19.06	0.00	5.65	0.00	0.00	0.24	0.00	0.00	0.00	1.65	2.59	0.94	3.29	0.00	0.0	0.00	0.0
I-B	1.48	94.15	0.15	2.10	0.03	0.03	0.15	0.00	0.15	0.03	0.79	0.38	0.41	0.06	0.00	0.0	0.12	0.0
I-C	0.00	0.26	98.75	0.00	0.00	0.00	0.23	0.00	0.00	0.10	0.31	0.13	0.16	0.05	0.00	0.0	0.00	0.0
I-D	4.48	12.24	0.17	76.55	0.00	0.00	0.00	0.00	0.00	0.00	4.83	1.55	0.00	0.17	0.00	0.0	0.00	0.0
I-E	0.00	0.03	0.01	0.00	99.71	0.02	0.00	0.09	0.00	0.01	0.04	0.06	0.00	0.03	0.00	0.0	0.00	0.0
I-F	0.00	0.10	0.03	0.00	0.00	99.53	0.00	0.03	0.00	0.10	0.03	0.07	0.07	0.00	0.00	0.0	0.03	0.0
I-U	1.54	0.00	20.00	0.00	0.00	0.00	36.92	0.00	0.00	0.00	23.08	13.85	0.00	4.62	0.00	0.0	0.00	0.0
II-A	0.00	0.03	0.03	0.00	0.06	0.03	0.00	96.69	0.00	2.89	0.23	0.00	0.00	0.00	0.00	0.0	0.06	0.0
II-B	0.00	0.49	0.49	0.00	0.49	0.00	0.00	0.49	94.63	0.49	1.46	0.00	0.00	1.46	0.00	0.0	0.00	0.0
II-C	0.00	0.04	0.04	0.00	0.00	0.08	0.00	3.96	0.00	95.38	0.23	0.04	0.08	0.08	0.00	0.0	0.08	0.0
III-A	0.62	4.38	0.68	1.93	0.00	0.00	0.17	1.36	0.06	0.74	79.55	6.02	0.85	3.47	0.06	0.0	0.11	0.0
III-B	2.60	4.25	0.96	1.23	0.14	0.00	0.27	0.00	0.00	0.00	17.67	63.56	0.00	9.18	0.14	0.0	0.00	0.0
III-C	6.67	30.00	0.00	0.00	0.00	3.33	0.00	0.00	3.33	3.33	23.33	1.67	16.67	8.33	0.00	0.0	3.33	0.0
III-D	4.25	2.00	0.25	1.25	0.00	0.00	0.75	0.00	1.00	1.00	26.00	25.25	2.25	34.75	0.75	0.0	0.00	0.5
III-E	0.00	0.00	0.00	0.00	0.00	0.00	0.00	0.00	0.00	0.00	20.00	0.00	0.00	80.00	0.00	0.0	0.00	0.0
IV-A	0.00	0.00	0.00	0.00	80.00	0.00	0.00	0.00	0.00	0.00	0.00	0.00	0.00	0.00	0.00	20.0	0.00	0.0
V-A	0.00	1.76	0.59	1.18	0.00	0.59	0.00	1.18	0.00	0.59	2.35	0.00	1.18	0.00	0.00	0.0	90.59	0.0
VI-A	0.00	0.00	0.00	0.00	0.00	0.00	0.00	0.00	0.00	0.00	0.00	0.00	0.00	80.00	20.00	0.0	0.00	0.0

Table S3: Runtime and RAM usage comparison. We selected a set of 650 genomes and achieved the following results using an Intel i5 machine with 8 GB of RAM.

Tool	CPU runtime	RAM usage
Casboundary	7 Hrs 23 Sec	5.3 MB
CRISPRCasFinder	3 Hrs 58 Sec	2.2 MB
